# Implementation of a food insecurity screening and referral program in student-run free clinics in San Diego, California

**DOI:** 10.1016/j.pmedr.2016.12.007

**Published:** 2016-12-08

**Authors:** Sunny Smith, David Malinak, Jinnie Chang, Maria Perez, Sandra Perez, Erica Settlecowski, Timothy Rodriggs, Ming Hsu, Alexandra Abrew, Sofia Aedo

**Affiliations:** a Department of Family Medicine and Public Health, University of California San Diego (UCSD), 9500 Gilman Drive #0696, La Jolla, CA 92093-0696, USA; b University of California San Diego (UCSD) School of Medicine, 9500 Gilman Drive #0696, La Jolla, CA 92093-0696, USA; c University of California San Diego (UCSD) Student-Run Free Clinic Project, 9500 Gilman Drive #0696, La Jolla, CA 92093-0696, USA

**Keywords:** AAP, American Academy of Pediatrics, ADA, American Diabetes Association, SNAP, Supplemental Nutrition Assistance Program, SRFC, Student-Run Free Clinic, SRFCP, Student-Run Free Clinic Project, UCSD, University of California San Diego, USDA, United States Department of Agriculture, Food insecurity, Food supply, Hunger, Medical students, Primary Health Care, Student-run Free Clinic

## Abstract

Food insecurity is associated with many poor health outcomes yet is not routinely addressed in clinical settings. The purpose of this study was to implement a food insecurity screening and referral program in Student-run Free Clinics (SRFC) and to document the prevalence of food insecurity screening in this low-income patient population. All patients seen in three SRFC sites affiliated with one institution in San Diego, California were screened for food insecurity using the 6-item United States Department of Agriculture (USDA) Food Security Survey between January and July 2015 and referred to appropriate resources. The percentage of patients who were food insecure was calculated. The screening rate was 92.5% (430/463 patients), 74.0% (318/430) were food insecure, including 30.7% (132/430) with very low food security. A food insecurity registry and referral tracking system revealed that by January 2016, 201 participants were receiving monthly boxes of food onsite, 66 used an off-site food pantry, and 64 were enrolled in the Supplemental Nutrition Assistance Program (SNAP). It is possible to implement a food insecurity screening and referral program into SRFCs. The prevalence of food insecurity in this population was remarkably high yet remained largely unknown until this program was implemented. Other health care settings, particularly those with underserved patient populations, should consider implementing food insecurity screening and referral programs.

## Highlights

• Food insecurity screening was successfully implemented into Free Clinics.
• Screening revealed a high prevalence of previously unaddressed food insecurity.
• A food insecurity screening and referral tracking system was implemented.
• More clinical settings should consider food insecurity screening and referrals.

## 1 Introduction

Food insecurity is an “economic and social condition of limited or uncertain access to adequate food” (United States Department of Agriculture: Economic Research Service, 2016). The United States Department of Agriculture (USDA) further describes food insecurity as “limited or uncertain availability of nutritionally adequate and safe foods or limited or uncertain ability to acquire acceptable foods in socially acceptable ways” (Life Sciences Research Office & Anderson, 1990). There are various length survey instruments used by the USDA to measure food insecurity, which include a 10-item tool and an expanded 18-item tool utilized for households with children (United States Department of Agriculture Economic Research Se, Bickel et al., 2000). A 2014 population study surveying over 43,000 households utilizing the 10-or 18-item screen as indicated based on household members estimated that 14.0% of households, or a projected 48 million people in the United States, were food insecure (United States Department of Agriculture Economic Research Service, 2016). The prevalence of food insecurity is higher in households with children (19.2%) as well as those headed by Hispanics (22.4%), and Blacks (26.1%) (Coleman-Jensen et al., 2015). The highest prevalence of food insecurity is seen in households headed by single mothers (35.3%) (Coleman-Jensen et al., 2015). The American Academy of Pediatrics (AAP) recently released a policy statement “Promoting Food Security for All Children” (Council on Community Pediatrics, 2015). This statement urges clinicians to screen all children for food insecurity, not just those in underserved communities, as many middle class families are also vulnerable to food insecurity with small changes in income (Council on Community Pediatrics, 2015). Appropriate referrals to food resources include local food pantries, Supplemental Nutrition Assistance Program (SNAP, formerly known as food stamps), Women Infants and Children (WIC), and free or reduced-price school lunch programs (Council on Community Pediatrics, 2015).

Adverse health consequences of inadequate access to food are apparent throughout the lifespan. Insufficient resources for food leads to individuals developing poor dietary habits and choosing less expensive, more filling, less healthy food options (Drewnowski, 2010, Rao et al., 2013). Analyses of data from the National Health Examination and Nutrition Examination Survey (NHANES) reveal that food insecurity is associated with hypertension, hyperlipidemia, and diabetes (Seligman et al., 2010, Seligman et al., 2007). Food insecurity is an independent risk factor for poor glycemic control in diabetes and nearly half of diabetics in safety-net clinics were food insecure (Seligman et al., 2012). The American Diabetes Association (ADA) recently added a section on managing food insecure patients to their Standards of Medical Care in Diabetes 2016 (American Diabetes Association Standards of Medical Care in Diabetes, 2016). The ADA described that patients with limited access to food are at risk for hyperglycemia as well as hypoglycemia, and recommended that providers seek local resources to help patients obtain nutritious foods (American Diabetes Association Standards of Medical Care in Diabetes, 2016). Feeding America, the nation's largest hunger relief agency, found that over two-thirds of their clients had to choose between paying for food or medical care within the last year (Weinfield et al., 2014). While health care providers do not routinely screen for food insecurity, most are willing to use a standardized screening instrument (Hoisington et al., 2012). Routine screening is an underutilized tool to address food insecurity, as food insecurity is often not readily apparent during clinical visits (Hoisington et al., 2012). In light of recent national guidelines changes, it is timely and pertinent for health care providers to consider systematically screening for food insecurity and referring to local resources in a broad range of settings, particularly those serving the underserved.

Student-run Free Clinics (SRFCs) are now present at over 75% of medical schools in the United States (Smith et al., 2014a). Like most SRFCs, the University of California San Diego (UCSD) Student-run Free Clinic Project (SRFCP) serves patients who are uninsured and unable to access care through the traditional health care safety-net. The UCSD SRFCP has previously been described in detail (Beck, 2005, Smith et al., 2014b). All patients are screened for eligibility, do not qualify for other health care programs including Medicaid, and are unable to afford even the low sliding-scale fees of community health centers. Our patient population is largely Latino and monolingual Spanish speaking. The UCSD SRFCP includes an interdisciplinary team that routinely involves social workers and social work interns. However, we had not systematically assessed food security in our patients, nor made routine food resource referrals until this program began.

This study was conducted to implement a food insecurity screening and referral program within the UCSD SRFCP and document the prevalence of food insecurity in this patient population.

## 2 Methods

This cross-sectional food insecurity screening study was conducted from January through July 2015. Outcomes of referrals to appropriate resources were documented through January 2016.

### 2.1 Study population

We screened all patients over 18 years of age seen for a medical visit at the Downtown San Diego, Pacific Beach, and South East San Diego sites of the UCSD SRFCP. There were further no exclusion criteria.

### 2.2 Survey instrument and survey administration

**Fig. 1:**
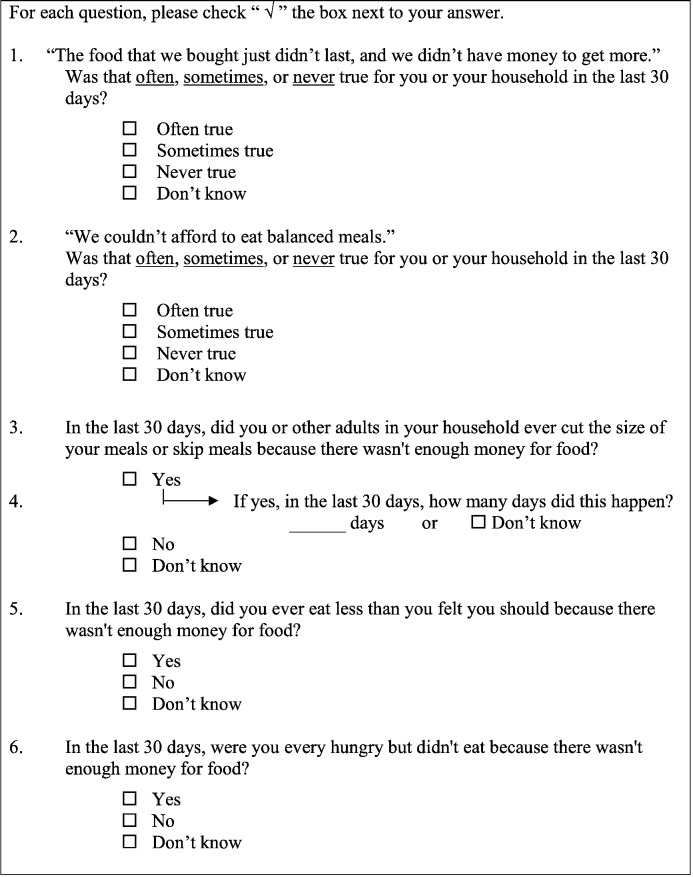
United States Department of Agriculture (USDA) US Household Food Security Survey 6-item screening tool used for the University of California San Diego (UCSD) Student-run Free Clinic Project (SRFCP) food insecurity screening and referral program at three clinic sites from January–July 2015.

We assessed food insecurity with the 6-item USDA US Household Food Security Survey, 30-day version (See Fig. 1) (United States Department of Agriculture Economic Research Se, Bickel et al., 2000). This tool is commonly used in research conducted on food insecurity in clinical settings (Seligman et al., 2012, Seligman et al., 2015, Moreno et al., 2015, Burkhardt et al., 2012). The 6-item survey has been found to be an acceptable alternative to the longer surveys as it correctly categorizes 97.7% of households when compared to the longer 10-item and 18-item formats (United States Department of Agriculture Economic Research Se, Bickel et al., 2000, Blumberg et al., 1999). The 6-item survey is intended to be filled out by an individual who represents the household, as the first four questions are constructed to ask about the household while the last two questions are targeted toward the individual (United States Department of Agriculture Economic Research Se, Bickel et al., 2000). Pre-health professional volunteer study coordinators handed surveys to patients immediately after check-in. The USDA provides this form in both English and Spanish and we offered surveys to patients in their preferred language. If patients expressed the need for assistance in filling out the form for any reason, including difficulty with literacy or vision, trained bilingual study volunteers offered assistance. Completed surveys were returned to study coordinators.

### 2.3 Scoring surveys

The USDA Food Security survey is scored on a scale of 0 to 6, with a score of 0–1 indicating high or marginal food security, 2–4 indicating low food security, and a score of 5–6 indicating very low food security (United States Department of Agriculture Economic Research Se, Bickel et al., 2000). High food security refers to individuals who have no food-access limitations. Marginal food security refers to those who often have anxiety over food shortages but do not tend to experience altered eating habits or diminished intake (United States Department of Agriculture Economic Research Se, Bickel et al., 2000). In contrast, low food security typically describes individuals who have reduced variety or quality of diet without reduced food intake, while very low food security typically describes both reductions in variety or quality as well as food intake (United States Department of Agriculture Economic Research Se, Bickel et al., 2000). Individuals with a score of 2–6 are considered to be food insecure according to USDA definitions (United States Department of Agriculture Economic Research Se, Bickel et al., 2000).

### 2.4 Referrals

After study coordinators received completed food security surveys, they provided all patients with information regarding local food pantries based on their home addresses. Resources were provided even if participants were not currently food insecure, as food insecurity is often episodic. Study coordinators asked patients if they had any concerns, tried to decrease stigma associated with not having enough food, explored common barriers to utilizing food resources, including food pantries, and answered questions. Study volunteers then verbally assessed patients to determine if they met eligibility criteria for SNAP based on immigration status, family income, household size, and current government assistance. They provided information on applying for SNAP benefits, if eligible. To decrease barriers to SNAP application, the UCSD SRFCP partnered with the County of San Diego, Feeding San Diego, San Diego Hunger Coalition, and Third Avenue Charitable Organization to initiate a pilot program to allow for same-day SNAP enrollment onsite monthly, in addition to providing the traditional two-step application process onsite regularly. If patients had diabetes, they were also offered the opportunity to receive monthly food distributions onsite as part of a new program to provide diabetes-appropriate nutritious foods. A predetermined study outcome included assessing if any differences existed in the prevalence of food insecurity in patients with and without diabetes in this population. Diabetes status was confirmed by checking the Problem List of the Electronic Health Record.

### 2.5 Addressing food insecurity as part of routine medical visits on an individual and systems-based level

Brief educational sessions were offered for medical students, residents, and faculty on food insecurity, its impact on health, and the importance of screening and referral. During routine visits, trainees or faculty were informed of their patients' food insecurity screening results, asked to address access to food, then record food insecurity status and the referral plan in the Electronic Health Record. They were instructed how to add food insecurity to the Problem List and the medical note, including in the Assessment and Plan, to facilitate follow up at subsequent visits. During daily clinic announcements, medical students, attending physicians, interdisciplinary students and faculty, including social workers, were regularly reminded to address food insecurity during clinic visits. A secure online spreadsheet was created as a patient registry that allowed study volunteers to follow up on referrals on an individual and population-level. These volunteers followed up with patients at each subsequent medical visit to assess if patients had gone to food pantries or received SNAP benefits. They tried to identify perceived barriers and help continually encourage patients connect with available food resources. Study volunteers populated the registry manually after direct interaction with patients during each clinic.

### 2.6 Evaluation of food insecurity screening and referrals in health care as a public health intervention

We assessed this project using the reach, effectiveness, adoption, implementation, and maintenance (RE-AIM) framework (Glasgow et al., 1999). This framework is designed to capture data needed to enhance the quality and public health impact of efforts to translate research into practice (RE-AIM. Reach Effectiveness Adoption Implementation Maintenance, 2016).

### 2.7 Data analysis

We summarized patient demographics using descriptive statistics, including means and standard deviations for continuous variables, and percentages for categorical variables. We determined the percent of patients with food insecurity (USDA Food Security Survey score 2–6), low food security (score 2–4), and very low food security (score 5–6). We used the Chi-squared test to compare the categories of food security status between groups. We summarized the number of patients who had received food resources as tracked in the patient registry. We calculated the number of health care professionals present using course rosters and clinic schedules. The UCSD Institutional Review Board approved this project.

## 3 Results

### 3.1 Screening rate and patient demographics

We screened 92.5% (430/463) of all patients for food insecurity. No patients refused to participate, and all were able to complete the survey themselves or with assistance from pre-health professional volunteers in either English or Spanish. Patient demographics are listed in Table 1. The mean age was 51.2 (SD 11.4) years old. The majority of patients were Latinos (420/430; 97.7%). Non-Latinos included 7 Caucasians (1.6%), 2 Asians (0.5%), and 1 Black (0.2%). Most of the patients were female (318/430: 74.0%). Nearly half of patients had diabetes (208/430; 48.4%). There were no differences in age, gender, race, or diabetes status between the three clinic sites.

### 3.2 Food insecurity prevalence

When including all three sites, 74.0% (318/430) of UCSD SRFCP patients screened were food insecure, including 30.7% (132/430) who had very low food security (Table 2). The prevalence of food insecurity ranged from 65.9% (112/170) at the South East San Diego Elementary School site, to 72.3% (73/101) at the Pacific Beach site, and 83.6% (133/159) at the downtown San Diego clinic site (*p* < 0.001) (Table 2). A higher percentage of patients with diabetes were food insecure (82.7%; 172/208) than those without diabetes (65.7%; 146/222) (*p* < 0.001) (Table 2).

### 3.3 Utilization of local food resources and government assistance

Study coordinators documented that Feeding San Diego provided monthly boxes of nutritious foods onsite for 201 patients with diabetes, 66 patients had obtained food from an off-site food pantry, and 64 patients were receiving SNAP.

### 3.4 Health care providers

At least 112 medical students, 42 faculty physicians, 18 residents, 1 physician assistant, 2 social workers, 4 social work interns, 3 community health promoters participated in clinical care during the study time-frame and were encouraged to discuss food insecurity with their patients. Health care trainees and providers received food insecurity screening results for their patents, incorporated assessing access to food as a part of routine health care visits, documented food insecurity in the Electronic Health Record, and followed up on referrals at subsequent visits.

### 3.5 Program assessment and potential public health impact

The reach, effectiveness, adoption, implementation, and maintenance of this program were analyzed using the RE-AIM framework summarized in Table 3 (Glasgow et al., 1999, RE-AIM. Reach Effectiveness Adoption Implementation Mainten).

## 4 Discussion

This study documents the implementation of a food insecurity screening and referral program for low-income patients at three different SRFC sites. Nearly all patients were successfully screened for food insecurity over a 6-month period. Previous studies in underserved safety-net clinics have documented food insecurity prevalence as high as 46% (Seligman et al., 2012). Nearly three-quarters of patients in this study reported food insecurity, with the range of 66% to 83% within the three clinic sites. To the authors' knowledge, this is the highest prevalence of food insecurity documented in a primary care setting to date. This data suggests that food insecurity is likely quite prevalent in underserved settings. SRFCs may be serving a particularly disadvantaged population, yet national data on food insecurity indicate that this problem affects approximately one in six people in the general population, including over a third of single mothers (Coleman-Jensen et al., 2015).

This study highlights the importance of screening for food insecurity, particularly in underserved populations, as it is likely under-recognized, under-diagnosed, and under-treated. Even with the very high level of food insecurity seen in this population, UCSD SRFCP's clinic's history of routinely addressing social determinants of health, and the availability of social workers on site, food insecurity was not an issue often discussed during medical visits before the implementation of this project.

Awareness of food insecurity, its effects on health, and the need for screening is likely to increase with the recent release of the AAP policy statement on promoting food security (Council on Community Pediatrics, 2015). They highlight the need for advocacy and to focus on medical education to teach about the health consequences of food insecurity (Council on Community Pediatrics, 2015). Since the majority of medical students now participate in SRFCs during their education (Smith et al., 2014a), SRFCs may be an ideal setting in which students can be empowered to implement food insecurity screening and referral programs, alongside interdisciplinary partners.

Pediatric, Family Medicine, Internal Medicine, and Primary Care clerkships and residencies also provide excellent opportunities to educate future physicians regarding the importance of screening for food insecurity and to role model these behaviors. Continuing Medical Education programs are needed to reach practicing physicians.

Screening for food insecurity takes little time and can be done by self-administered patient questionnaires. This study utilized the 6-item USDA survey that is often used in clinical research as it allows for documenting the severity of food insecurity, however an even shorter survey is available (Seligman et al., 2012, Hager et al., 2010). A simple two-item screening questionnaire is considered easiest for use in clinical practice and is commonly recommended as it has been shown to have a 97% sensitivity when compared with the longer USDA surveys (Hager et al., 2010). A positive response to either item is considered food insecure.

Physicians, dietitians, and nutritionists often counsel patients on the benefits of changing their diet to lose weight and improve control of their chronic health conditions. However, much of the advice, such as increasing fresh fruits and vegetables may be perceived as impractical for those who are most food insecure. Providers need to be educated on how to counsel patients to eat healthy foods on a limited budget and what resources are available. Social workers and community health workers may address food insecurity or other social determinants of health with patients as a result of screening conducted in a busy primary care setting (Page-Reeves et al., 2016). Electronic Medical Records can be used to trigger a reminder for screening and generate referrals.

The patient registry created as a result of this program allowed us to begin to follow not only food insecurity status, but if patients followed through with food pantry resources, and if they received SNAP benefits. We began a dialogue with patients to explore perceived barriers to visiting a food pantry or enrolling in government food assistance programs and attempted to address these barriers. However, utilization of off-site resources was still low. We are now partnering with Feeding San Diego to provide nutritious food distributions to all patients on-site at the UCSD SRFCP.

Recording food insecurity screening results into the medical record enabled us to address food insecurity as an ongoing issue in medical care. This project allowed nearly 200 health care providers and trainees to learn to address food insecurity. This is a skill that can be applied to initiate conversations regarding access to food, even when working in other inpatient or outpatient health care settings that do not include a systems-based approach to food insecurity.

Patients with diabetes had a higher prevalence of food insecurity than patients without diabetes in this study. Cyclic access to food has been linked to increased hospitalizations for hypoglycemia in the poor toward the end of the month as food supplies run out (Seligman et al., 2014). Many low-income households have varying access to food that changes based on number of hours worked or government assistance that is typically received at the beginning of each month. Patients are also often faced with the difficult decision of choosing to pay for food or medication in resource poor settings (Weinfield et al., 2014). Addressing food insecurity and other social determinants of health may one day become a part of the routine social history during medical visits as standard as assessing for alcohol, tobacco, or drug use.

This study has several limitations. Patient-administered surveys relied on self-report to assess food insecurity. Results may be inaccurate due to recall-bias, education-level, literacy barriers, influenced by shame, or preference not to discuss with a health care provider. However, we used the USDA 6-item survey to standardize our data with other large published data sets and provided assistance to anyone requesting help. Although conducted at three separate clinic sites, all were SRFCs, in one city, affiliated with one institution in San Diego, California, with a predominantly Latino patient population. Similar food insecurity prevalence may not be found at other low-income clinics across the country. However, based on national data and current policy statements, it is likely that screening for food insecurity would be useful in other settings. We did not determine directionality or evaluate confounding factors with regard to the association between food insecurity and diabetes. Finally, we have not yet determined if patients are less food insecure as a result of this project. This remains an area for further inquiry.

Other future areas of study could include examining if the level of food insecurity is correlated with health outcomes in this population, assessing the impact of this program on medical student and provider knowledge, skill, attitudes, documentation, and referral patterns. Multi-institutional studies are needed to examine generalizability. However, presentation of this data has resulted in the implementation of additional food insecurity screening programs in other low-income clinics.

In conclusion, implementing food insecurity screening and referral programs can serve as a useful tool in determining and addressing food insecurity within a clinical setting. Systematic food insecurity screening and referrals should be considered in SRFCs, in other medical education settings, and more broadly in health care settings, particularly in underserved practices including community health centers who serve those most likely to be food insecure.

## Conflict of interest

No financial disclosures were reported by the authors of this paper.

## Funding/support

This research did not receive any specific grant from funding agencies in the public, commercial, or not-for-profit sectors.

## Prior presentations

Prior versions of this data were presented at the University of California San Diego Public Health Research Day La Jolla, California April 2015, American Association of Family Physicians National Conference of Family Medicine Residents and Medical Students Kansas City, Missouri August 2015, Network of Ethnic Physician Organizations and California Medical Association Building Healthy Communities Summit, Riverside, California September 2015, and Society of Student-run Free Clinics in Phoenix, Arizona, February 2016.

## Ethical approval

The University of California San Diego Institutional Review Board approved this project 141481.

## Figures and Tables

**Table 1:** Demographics of food insecurity screening and referral program participants at three sites of the University of California San Diego (UCSD) Student-run Free Clinic Project (SRFCP) from January–July 2015.

	All patients *N* = 430	Downtown *N* = 159	South East San Diego *N* = 170	Pacific Beach *N* = 101	P value
	Mean (SD)	Mean (SD)	Mean (SD)	Mean (SD)	
Age in years,	51.3 (11.4)	52.1 (11.0)	50.63 (12.0)	51.3 (11.2)	0.55

	n(%)	n(%)	n(%)	n(%)	
Male	112 (26.0%)	43 (27.0%)	36 (21.2%)	33 (32.7%)	0.11
Female	318 (74.0%)	116 (73.0%)	134 (78.8%)	68 (67.3%)	
Latino	420 (97.7%)	154 (96.9%)	169 (99.4%)	97 (96.0%)	0.14
Non-Latino	10 (2.3%)	5 (3.1%)	1 (0.6%)	4 (4.0%)	
Diabetic	208 (48.4%)	82 (51.6%)	82 (48.2%)	44 (43.6%)	0.45
Non-diabetic	222 (51.6%)	77 (48.4%)	88 (51.8%)	57 (56.4%)	

**Table 2:** Results from the food insecurity screening and referral program at three sites of the University of California San Diego (UCSD) Student-run Free Clinic Project (SRFCP) from January–July 2015. Utilizing the United States Department of Agriculture (USDA) US Household Food Security Survey 6-item screening tool. Comparison of prevalence in patients with diabetes to those without diabetes and by clinical site.

	All patients *N* = 430	Patients with diabetes *N* = 208	Patients without diabetes *N* = 222	P-value	Downtown *N* = 159	South East San Diego *N* = 170	Pacific Beach *N* = 101	P-value
Food secure (score 0–1), n (%)	112 (26.0%)	36 (17.3%)	76 (34.2%)	< 0.001	26 (16.4%)	58 (34.1%)	28 (27.7%)	< 0.001
Food insecure (score 2–6), n (%)	318 (74.0%)	172 (82.7%)	146 (65.8%)		133 (83.6%)	112 (65.9%)	73 (72.3%)	
Low Food security (score 2–4), n (%)	186 (43.3%)	98 (47.1%)	88 (39.6%)		68 (42.8%)	74 (43.5%)	44 (43.6%)	
Very low food security (score 5–6), n (%)	132 (30.7%)	74 (35.6%)	58 (26.1%)		65 (40.9%)	38 (22.4%)	29 (28.7%)	

**Table 3:** Analysis of the food insecurity screening and referral program at three sites of the University of California San Diego (UCSD) Student-run Free Clinic Project (SRFCP) from January–July 2015 using the RE-AIM framework (Reach, Effectiveness, Aim, Implementation, and Maintenance).

RE-AIM element	Outcome
Reach	
Exclusion criteria	None
Percent individuals who participated	92.5% (430/463 patients screened)

Effectiveness	
Measure of primary outcome: Food insecurity	74.0% (318/430 of patients screened) were food insecure, including 30.7% (132/430) with very low food security
Measure of broader outcomes: Utilization of referral to food resources	201 received boxes of nutritious food onsite 66 used an off-site food pantry, 64 enrolled in the Supplemental Nutrition Assistance Program (SNAP)

Adoption	
Setting Exclusions	None
Percent of settings approached that participated	100% (3/3)
Characteristics of settings participating	Three Student-run Free Clinics in San Diego, California serving a low-income, uninsured, largely Latino patient population
Utilization of food insecurity registry	92.5% (430/463) of patients seen had food insecurity screening results entered into the registry

Implementation	
Percent of perfect delivery, adaptations made to intervention	The intervention was delivered as intended, no known adaptations were made.
Cost of intervention	There were no costs to screening and making referrals as volunteer staff performed screening, referrals, tracking, and follow-up.
Consistency of implementation across staff, settings, subgroups	No known inconsistencies.

Maintenance	
Long term attrition	Volunteers are conducting follow-up screening for food insecurity at all three sites and patients have not refused to fill out follow-up surveys.
If program is still ongoing at least 6 months post study	Follow-up food insecurity screening and referrals, including on-site food distributions, are being provided over one year after initial study completion.
If and how program was adapted long term	This program has grown to routine food insecurity screening every 6 months. Partnership with a local food bank, Feeding San Diego, was developed to allow distribution of healthy food on-site to all patients.
Alignment of organization mission or sustainability	Pre-existing mission statements of UCSD Student-run Free Clinic Project and Feeding San Diego are well-aligned with addressing food insecurity in health care. Both organizations are committed to sustainability of this project.

## References

[bb0005] American Diabetes Association Standards of Medical Care in Diabetes (2016). Diabetes Care.

[bb0010] Beck E. (2005). The UCSD student-run free clinic project: transdisciplinary health professional education. J. Health Care Poor Underserved.

[bb0015] Bickel G., Nord M., Price C., United States Department of Agriculture (2000). Measuring Food Security in the United States, Revised 2000. U.D.

[bb0020] Blumberg S.J., Bialostosky K., Hamilton W.L. (1999). The effectiveness of a short form of the household food security scale. Am. J. Public Health.

[bb0025] Burkhardt M.C., Beck A.F., Conway P.H. (2012). Enhancing accurate identification of food insecurity using quality improvement techniques. Pediatrics.

[bb0030] Coleman-Jensen A., Rabbitt M.P., Gergory C. (2015). Household Food Security in the United States in 2014. (Economic Research Report No. ERR-194). http://www.ers.usda.gov/media/1896841/err194.pdf.

[bb0035] Council on Community Pediatrics (2015). Promoting food security for all children. Pediatrics.

[bb0040] Drewnowski A. (2010). The cost of US foods as related to their nutritive value. Am. J. Clin. Nutr..

[bb0045] Glasgow R.E., Vogt T.M., Boles S.M. (1999). Evaluating the public health impact of health promotion interventions: the RE-AIM framework. Am. J. Public Health.

[bb0050] Hager E.R., Quigg A.M., Black M.M. (2010). Development and validity of a 2-item screen to identify families at risk for food insecurity. Pediatrics.

[bb0055] Hoisington A.T., Braverman M.T., Hargunani D.E. (2012). Health care providers' attention to food insecurity in households with children. Prev. Med..

[bb0060] Life Sciences Research Office, Anderson S.A. (1990). Core indicators of nutritional state for difficult-to-sample populations. J. Nutr..

[bb0065] Moreno G., Morales L.S., Isiordia M. (2015). Latinos with diabetes and food insecurity in an agricultural community. Med. Care.

[bb0070] Page-Reeves J., Kaufman W., Bleeker M. (2016). Addressing social determinants of health in a clinic setting: the WellRx pilot in Albuquerque, New Mexico. J. Am. Board Fam. Med..

[bb0075] Rao M., Afshin A., Singh G. (2013). Do healthier foods and diet patterns cost more than less healthy options? A systematic review and meta-analysis. BMJ Open.

[bb0080] RE-AIM. Reach Effectiveness Adoption Implementation Maintenance (2016). http://re-aim.org.

[bb0085] Seligman H.K., Bindman A.B., Bittinghoff E. (2007). Food insecurity is associated with diabetes mellitus: results from the National Health Examination and Nutrition Examination Survey (NHANES) 1999–2002. J. Gen. Intern. Med..

[bb0090] Seligman H.K., Laria B.A., Kushel M.B. (2010). Food insecurity is associated with chronic disease among low-income NHANES participants. J. Nutr..

[bb0095] Seligman H.K., Tschann J., Jacobs E.A. (2012). Food insecurity and glycemic control among low-income patients with type 2 diabetes. Diabetes Care.

[bb0100] Seligman H.K., Bolger A.F., Guzman D. (2014). Exhaustion of food budgets at month's end and hospital admissions for hypoglycemia. Health Aff..

[bb0105] Seligman H.K., Lyles C., Marshall M.B. (2015). A pilot food bank intervention featuring diabetes-appropriate food improved glycemic control among clients in three states. Health Aff..

[bb0110] Smith S., Thomas R., Cruz M. (2014). Presence and characteristics of student-run free clinics in medical schools. JAMA.

[bb0115] Smith S.D., Yoon R., Johnson M.L. (2014). Effect of involvement in a student-run free clinic project on attitudes toward the underserved and interest in primary care. J. Health Care Poor Underserved.

[bb0120] United States Department of Agriculture Economic Research Service (2016). Survey tools. http://www.ers.usda.gov/topics/food-nutrition-assistance/food-security-in-the-us/survey-tools.aspx.

[bb0125] United States Department of Agriculture: Economic Research Service (2016). Food security in the United States: measurement. http://www.ers.usda.gov/topics/food-nutrition-assistance/food-security-in-the-us/measurement.aspx.

[bb0130] Weinfield N.S., Mills G., Borger C. (2014). Hunger in America 2014: National Report. Chicago: Feeding America. http://help.feedingamerica.org/HungerInAmerica/hunger-in-america-2014-full-report.pdf.

